# Re-expression of miR-200c suppresses proliferation, colony formation and *in vivo* tumor growth of murine claudin-low mammary tumor cells

**DOI:** 10.18632/oncotarget.15829

**Published:** 2017-03-02

**Authors:** Robert Jones, Katrina Watson, Anthony Bruce, Sarah Nersesian, Jenna Kitz, Roger Moorehead

**Affiliations:** ^1^ Department of Biomedical Science, Ontario Veterinary College, University of Guelph, Guelph, Ontario, Canada

**Keywords:** microRNA, miR-200f, miR-200c, mammary tumors, claudin-low

## Abstract

Claudin-low breast cancer is a relatively rare breast cancer subtype. These cancers are typically ER^−^/PR^−^/HER2^−^ and express high levels of mesenchymal genes as well as genes associated with inflammation, angiogenesis and stem cell function. In addition to alterations in gene expression, it was recently demonstrated that claudin-low breast cancers express very low levels of the miR-200 family of miRNAs. Given that each miRNA can regulate tens, hundreds or even thousands of genes, miRNAs are being evaluated as therapeutic targets. In this study we show that mammary tumors from MTB-IGFIR transgenic mice and cell lines derived from these tumors represent a model of human claudin-low breast cancer and murine claudin-low mammary tumors and cell lines express only very low levels of all five members of the miR-200 family. Reduced miR-200 family expression appears to be regulated via methylation as cells and tumors expressing low levels of miR-200 family members had higher levels of CpG methylation in a putative promoter region than tumors and cells expressing high levels of miR-200 family members. Re-expression of miR-200c in murine claudin-low mammary tumor cells inhibited tumor cell proliferation and colony formation *in vitro* and tumor growth *in vivo*. With respect to tumor growth *in vivo*, re-expression of miR-200c was associated with a reduction in tumor vasculature and expression of *Flt1* and *Vegfc*. Therefore, miR-200c is an important regulator of mesenchymal tumor cell growth.

## INTRODUCTION

MicroRNAs (miRNAs or miRs) are small non-coding RNA molecules approximately 19-22 nucleotides (nt) long [1, 2] that were originally identified in *C. elegans* in 1993 [3, 4]. Subsequent studies on miRNAs determined that most miRNAs are initially transcribed as long primary transcripts (pri-miRNA) ranging from hundreds to thousands of nucleotides in length [5, 6]. These pri-miRNAs are then processed in the nucleus by Drosha, a ribonuclease III endonuclease, resulting in a ~60-80 nt precursor transcript or pre-miRNA [5, 7, 8]. In the next step, pre-miRNAs are exported from the nucleus by Exportin 5 [8]. In the final step pre-miRNAs are cleaved into 19-22 nt double-stranded duplexes by another RNaseIII nuclease, Dicer [5, 9]. Mature miRNAs are incorporated into a ribonucleoprotein complex known as the RNA-induced silencing complex (RISC) [5]. Most miRNAs in mammals direct the RISC complex to target mRNAs and this complex binds to the 3′-UTRs of mRNAs using the seed region (nucleotides 2-8) of the miRNA [5, 7, 8, 10, 11]. RISC complex binding to target mRNAs typically induce translational repression and mRNA destabilization [5, 7, 8, 10]. Since only the seed region of miRNAs is required to bind mRNA, each miRNA can potentially regulate hundreds of mRNAs [12]. Several computational algorithms such as microRNA.org or TargetScan have now been developed that predict these potential mRNA targets [5]. Since there are over 2500 miRNAs identified in humans [13] and each miRNA can potentially regulate hundreds, or in some cases, thousands of mRNAs, miRNAs have been reported to regulate over 60% of the protein coding genes and thus represent one of the main classes of gene regulatory molecules in mammalian cells.

Given that miRNAs regulate gene expression it is not surprising they can play a role in cancer development. When aberrantly expressed in cancer, miRNAs can act as tumour suppressors that repress oncogenic mRNAs, or as oncogenes that repress tumour suppressor genes [12, 14]. One family of microRNAs that has garnered considerable attention in cancer biology is the miRNA-200 family (miR-200f) which consists of 5 members, miR-141, miR-200a, miR-200b, miR-200c and miR-429. This family of microRNAs is expressed as two clusters on distinct chromosomes with the miR-200c/miR-141 cluster located on chromosome 12 in humans and chromosome 6 in mice and the miR-200b/miR-200a/miR-429 cluster located on chromosome 1 in humans and chromosome 4 in mice [15]. The seed sequence, the region of the miRNA that determines mRNA binding, is the same in miR-200b, miR-200c, and miR-429 (AAUACUG). miR-200a and miR-141 share the same seed sequence (AACACUG) that is different from the seed sequence of miR-200b, miR-200c and miR-429 by one nucleotide [16]. Expression of the miR-200 clusters appears to be regulated by modifications to the promoter regions of each cluster. Promoter hypermethylation appears to be the primary mechanism for silencing miR-200c/141 expression while histone modifications via the Polycomb group has been reported to be responsible for silencing miR-200b/200a/429 expression [17].

The miR-200f regulates a number of properties important for cancer initiation and progression including epithelial-to-mesenchymal transition (EMT), proliferation, migration, and characteristics associated with stem/progenitor cells [13, 18–22]. Several studies have shown that miR-200f members negatively regulate mesenchymal transcription factors such as *Zeb1*, *Zeb2*, *Twist1*, *Twist2*, *Snai1*, and *Snai2*. These mesenchymal transcription factors repress E-cadherin transcription and genes that promote epithelial polarity [23–26] while inducing the expression of mesenchymal genes such as *Vim* and *S100A4* [27, 28]. Therefore, loss of miR-200f members results in cells taking on a more mesenchymal phenotype potentially leading to enhanced migratory ability, increased metastatic potential and poorer patient prognosis. Consistent with it's role in EMT, studies in breast cancer have shown that the miR-200f is expressed in human luminal A breast cancers (tumor cells have epithelial characteristics) but lost in triple negative breast cancers including the claudin-low subtype (tumor cells have mesenchymal characteristics) [29, 30].

Claudin-low tumors were first described by Prat et al [31] and represent a subtype of triple-negative breast cancer characterized by high levels of markers associated with EMT such as *Twist1*, *Twist2*, *Zeb1*, *Zeb2*, *Snai1*, and *Snai2* while expressing little or no markers of luminal differentiation [32]. In addition, claudin-low tumors express high levels of immune-related, angiogenesis and stem cell genes [31] and often display characteristics of metaplastic and medullary differentiation [33]. A subsequent study by Lehmann et al [34] identified 6 distinct subtypes of triple-negative breast cancer; basal-like 1, basal-like 2, immunomodulatory, mesenchymal, mesenchymal stem-like and luminal androgen receptor. The mesenchymal stem-like subtype displayed low levels of claudin-3, -4 and -7 and thus have similar characteristics to claudin-low tumors. Finally, Jézéquel et al [35] identified 3 subtypes of triple-negative breast cancer; luminal androgen receptor, basal-like with low immune response and high M2 macrophages, and basal-enriched with high immune response and low M2 macrophages. Most of the claudin-low tumors were assigned to the basal-enriched with high immune response and low M2 macrophage subtype [35]. Since the nomenclature most appropriate for describing the different subtypes of triple negative breast cancer remains unresolved [35], this manuscript will use the term claudin-low to be consistent with prior publications on the tumors and cell lines derived from MTB-IGFIR transgenic mice [36–40].

Since one of the hallmarks of claudin-low breast cancer is the suppression of miR-200f members, this manuscript evaluated the impact of re-expressing a miR-200f member, namely miR-200c, in murine mammary tumor cells with characteristics similar to human claudin-low breast cancer. Our findings indicate that miR-200c can suppress proliferation and colony formation of murine claudin-low tumor cells *in vitro* and impair tumor growth *in vivo*, potentially through inhibiting angiogenesis.

## RESULTS

### miRNA expression in WT, PMT and RST samples

A miRNA array was performed using an Agilent 8×15K v2:627 mouse miRNA array and 10 primary mammary tumor (PMT) samples and 6 recurrent spindle tumor (RST) samples. PMTs develop in transgenic mice due to elevated expression of the type I insulin-like growth factor receptor (IGF-IR) and these tumors have an epithelial appearance but cluster most closely with human basal-like breast cancers [39, 41, 42]. RSTs are mammary tumors that arise following downregulation of the IGF-IR transgene in established mammary tumors in MTB-IGFIR transgenic mice. RSTs have a mesenchymal morphology and cluster most closely with human claudin-low breast cancers [39, 41]. Detailed characterization of PMTs and RSTs have been previously described [39, 41, 42].

The top 15 differentially expressed microRNAs based on fold change (p<0.05) are presented in Table 1. The most differentially expressed miRNA was mmu-miR-429, a member of the miR-200 family, and 4 of the 5 miR-200f members (miR-141, miR-200b, miR-200c and miR-429) were in the top 15 differentially expressed miRNAs (shaded in Table 1). Quantitative RT-PCR was used to validate the differential expression of the miR-200 family and as shown in Table 2, all 5 members of the miR-200f (miR-200a, miR-200b, miR-200c, miR-141 and miR-429) were expressed at significantly higher levels in the PMT samples compared to the RST samples. The magnitude of the difference of miR-200f members in PMT samples compared to RST samples detected by qRT-PCR was much greater than that detected by the miRNA array. All miR-200f members were expressed at least 70-fold higher in the PMT samples compared to the RST samples as determined by qRT-PCR (Table 2).

**Table 1:** Top 15 microRNAs differentially expressed in PMTs vs RSTs based on fold change

microRNA	Fold Change PMT vs RST
mmu-miR-429	23.8
mmu-miR-290-5p	−20.8
mmu-miR-466f-5p	19.0
mmu-miR-148a	18.7
mmu-miR-685	18.0
mmu-miR-221	−17.4
mmu-miR-211	16.9
mmu-miR-16*	16.2
mmu-miR-503	−16.2
mmu-miR-146a	−16.1
mmu-miR-200c	14.6
mmu-miR-466h	13.1
mmu-miR-141	11.7
mmu-miR-690	−11.5
mmu-miR-200b	11.4

**Table 2:** miR-200f in PMTs and RSTs

miRNA^1^	PMT	RST^2^
miR-200a	1	9.9×10^−3^*
miR-200b	1	1.4×10^−2^*
miR-200c	1	6.7×10^−3^*
miR-141	1	4.6×10^−3^*
miR-429	1	1.4×10^−2^*

^1^normalized to SnoRNA202 and SnoRNA234.

^2^miRNA levels expressed relative to PMT.

*p<0.05.

### Expression of miR-200c in RJ345, RJ348 and RJ423 cells

**Figure 1:**
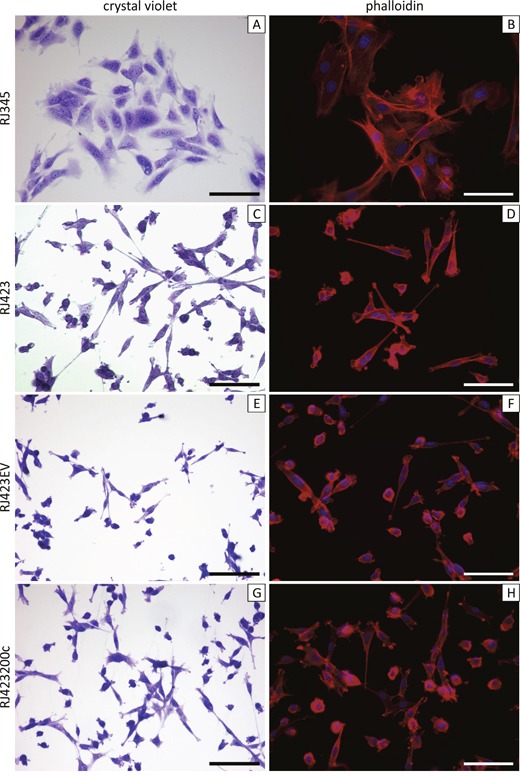
Representative images of crystal violet stained (**A,C,E,G**) and phalloidin stained (**B,D,F,H**). RJ345 (A,B), RJ423 (C,D), RJ423EV (E,F) and RJ423200c (G,H) cells. Scale bars, 100μm.

Cell lines have been generated from some of the PMTs and RSTs that developed in MTB-IGFIR transgenic mice. RJ345 cells were derived from a PMT and have epithelial morphology when grown as monolayers (Figure 1A, 1B), express the luminal epithelial gene *Cdh1* [43] and express only very low levels of mesenchymal genes such as *Twist1*, *Twist2*, *Zeb1*, and *Zeb2* [43]. In addition, this cell line is only weakly tumorigenic when injected into mammary fat pad of syngeneic FVB mice [43]. RJ348 cells were derived from a RST and have a mesenchymal morphology in culture, express low levels of *Cdh1* and high levels of *Twist1*, *Twist2*, *Zeb1*, *Zeb2* [43]. RJ348 cells are highly tumorigenic when injected into the mammary fat pad of syngeneic FVB mice [43]. One problem with the RJ348 cells is that they are difficult to stably transfect and thus another cell line with mesenchymal morphology, RJ423, was used in this study. RJ423 cells were derived from a different RST than RJ348 cells and RJ423 cells, like RJ348 cells have a mesenchymal morphology when grown as monolayer (Figure 1C, 1D).

Table 3 shows the expression of miR-200f members in RJ348 and RJ423 cells relative to RJ345 cells. RJ345 cells express all members of the miR-200f at levels significantly higher than either RJ348 or RJ423 cells. Therefore, the cell lines retain miR-200f expression profile of the tumors from which they were derived; both RJ345 and PMTs express high levels of miR-200f while RJ348, RJ423 and RSTs all express very low levels of miR-200f.

**Table 3:** miR-200f in RJ345, RJ348 and RJ423 cells

miRNA^1^	RJ345	RJ348^2^	RJ423^2^
miR-200a	1	2.8×10^−5^*	9.3×10^−6^*
miR-200b	1	3.7×10^−5^*	3.7×10^−5^*
miR-200c	1	3.7×10^−4^*	3.2×10^−4^*
miR-141	1	1.8×10^−4^*	8.4×10^−5^*
miR-429	1	ND^3^*	ND*

^1^miRNA expression normalized to SnoRNA202 and SnoRNA234.

^2^miRNA levels expressed relative to RJ345 cells.

^3^ND - not detected.

*p<0.05.

**Figure 2:**
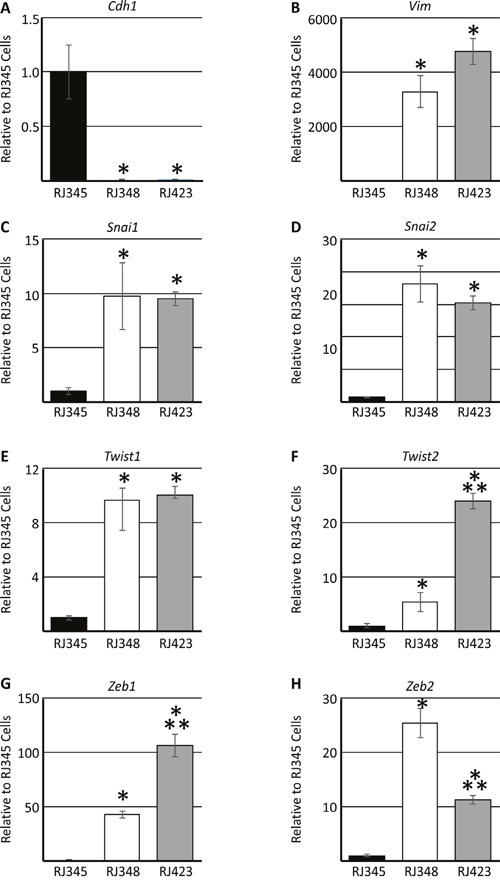
Expression (mean ± SEM) of E-cadherin/*Cdh1* **(A)**, vimentin/*Vim* **(B)**, Snail/*Snai1* **(C)**, Slug/*Snai2* **(D)**, *Twist1* **(E)**, *Twist2* **(F)**, *Zeb1* **(G)** and *Zeb2* **(H)** as determined by qRT-PCR. Gene expression in RJ348 and RJ423 cells are expressed relative to RJ345 cells. *Hprt* was used as the housekeeping gene and * indicates significant difference (p<0.05) in RJ348 and RJ423 cells compared to RJ345 cells while ** indicates significant differences (p<0.05) between RJ348 and RJ423 cells. Each cell line represents n=3.

Figure 2 shows the expression of selected epithelial (*Cdh1*; Figure 2A) and mesenchymal (*Snai1*, *Snai2*, *Twist1*, *Twsit2*, *Vim*, *Zeb1*, *Zeb2*; Figure 2B-2H) genes in RJ345, RJ348 and RJ423 cells. RJ348 and RJ423 both express high levels of all the mesenchymal genes and only very low levels of *Cdh1*. Although RJ348 and RJ423 cells were both derived from RSTs and have similar morphology in culture, the levels of some of the mesenchymal genes did vary in that RJ423 cells had significantly higher levels of *Twist2* and *Zeb1* than RJ348 cells (Figure 2F, 2G) while RJ348 cells had significantly higher levels of *Zeb2* than RJ423 cells (Figure 2H). Both RJ348 and RJ423 cells expressed only extremely low levels of *Cldn3*, *Cldn4* and *Cldn7* (data not shown) suggesting that both RJ348 and RJ423 cells share features of human claudin-low breast cancers.

### miR-200c is regulated by promoter methylation

**Figure 3:**
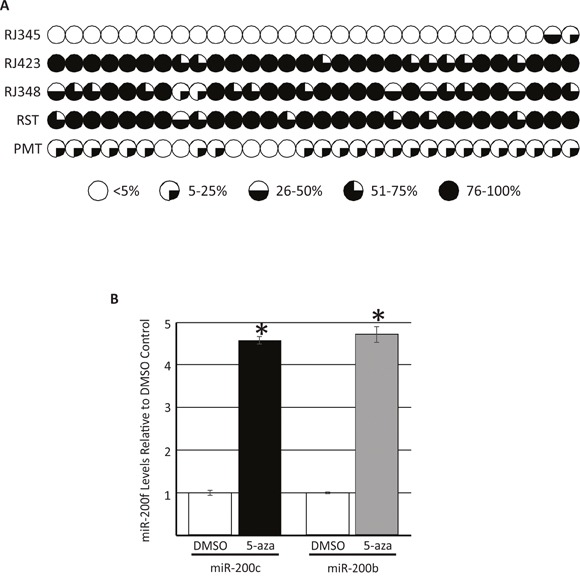
Evaluation of the methylation status of 30 CpG sites ~1000bp upstream of the miR-200c/miR-141 cluster on murine chromosome 6 as determined by targeted bisulfite sequencing Each CpG site is represented by an individual pie and percentage of CpG sites methylated are indicated by the amount of shading; <5% methylation, 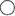, 5-25% methylation, 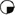, 26-50% methylation, 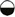, 51-75% methylation, 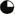, and >75% methylation, 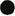 **(A)**. miR-200c and miR-200b (first miRNA in miR-200c/141 or miR-200b/200a/429 cluster) levels in RJ423 cells treated daily with DMSO or 3μM of the DNA methyltransferase inhibitor, 5-aza-2′-deoxycytidine (5-aza) for 72hrs **(B)**. Each bar represents the mean ± SEM of 3 independent trials, *p<0.05.

Previous reports indicate that the miR-200c/miR-141 promoter region can be methylated and this methylation decreases miR-200c and miR-141 expression [44–47]. To assess miR-200c promoter methylation in our model systems two approaches were employed. First, targeted bisulfite sequencing of a putative miR-200c/miR-141 promoter region was performed in RJ345, RJ423 and RJ348 cell lines as well as in PMT and RST samples. Thirty-three CpG sites within a region ~1000bp upstream of the miR-200c/miR-141 start site were evaluated. Of the 33 CpG sites, 30 CpG sites had at least 10 read counts in all three cell lines and tumor tissue. At each CpG site, the total CpG count and the methylated CpG count were determined and these values were used to calculate a methylated CpG ratio. Figure 3A shows the methylated CpG ratio of the 30 CpG sites with high read counts in a graphical representation where each individual pie represents a distinct CpG site. If the average CpG methylation ratio was <5% the pie appears completely white. Average CpG methylation ratio of 5-25% was indicated by one quarter of the pie being shaded black while CpG methylation ratios of 26-50% and 51-75% represent one half and three quarters of the pie being shaded black, respectively. If the CpG methylation ratio was >75% the entire pie was shaded black. As shown in Figure 3A the level of methylation was higher in RJ423 and RJ348 cells compared to RJ345 cells and 29/30 sites in RJ423 cells and 28/30 sites in RJ348 reached statistical significance. CpG methylation was also higher in RST samples compared to PMT samples and CpG methylation ratio was statistically higher in 29/30 of the sites.

In the second approach, RJ423 cells were treated with the DNA methyltransferase inhibitor, 5-aza-2′-deoxycytidine for 72 hours. Treatment of cells with 5-aza-2′-deoxycytidine has been shown to reduce DNA methylation and increase expression of microRNAs regulated by methylation [44]. Figure 3B shows the levels of the first member of each miR-200f cluster (miR-200c and miR-200b) in RJ423 cells treated with the vehicle control (DMSO) or 5-aza-2′-deoxycytidine. 5-aza-2′-deoxycytidine treatment significantly increased the expression of both miR-200c (4.6-fold) and miR-200b (4.7-fold) suggesting that the expression of both miR-200 clusters are regulated, at least in part, by methylation.

### Re-expression of miR-200c in RJ423 cells

To determine the function of miR-200f in murine mammary tumor cells with features of human claudin-low breast cancer, miR-200c was re-expressed in RJ423 cells. As mentioned above, RJ423 cells were selected as it is easier to stably transfect these cells than the RJ348 cells. We also chose to re-express only one miR-200f member to simplify the data analysis. It should be noted that although only miR-200c was re-expressed in RJ423 cells, miR-200c has hundreds of predicted mRNA targets (microrna.org, targetscan.org, mirdb.org).

**Figure 4:**
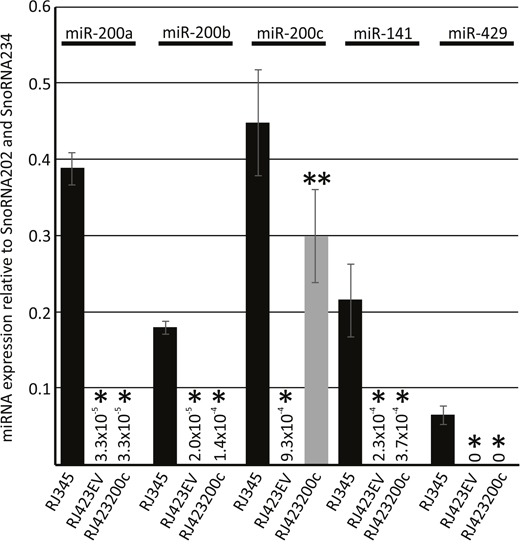
Expression (mean ± SEM) of miR-200a, miR-200b, miR-200c, miR-141 and miR-429 in RJ345 cells, RJ423 cells containing the empty vector control (RJ423EV) or RJ423 cells containing miR-200c (RJ423200c) as determined by Taqman RT-PCR The expression of each miRNA was normalized to the levels of SnoRNA202 and SnoRNA234. *indicates significant difference (p<0.05) in RJ4232EV and RJ423200c cells compared to RJ345 cells while **indicates significant differences (p<0.05) between RJ423EVand RJ423200c cells. Each cell line represents n≥3.

RJ423 cells were transfected with either the control plasmid (pCMV-MIR) creating RJ423EV cells or a plasmid expressing miR-200c driven by a CMV promoter (pCMV-MIR containing the miR-200c) creating RJ423200c cells. TaqMan RT-PCR revealed that the RJ423200c cells expressed significantly higher levels of miR-200c compared to RJ423EV cells (>300-fold increase in miR-200c levels) and the level of miR-200c expression in the RJ423200c cells was nearly restored to the levels expressed by RJ345 cells (Figure 4). None of the other miR-200f members were elevated in the RJ423200c cells indicating that miR-200c was specifically upregulated in these cells (Figure 4).

**Figure 5:**
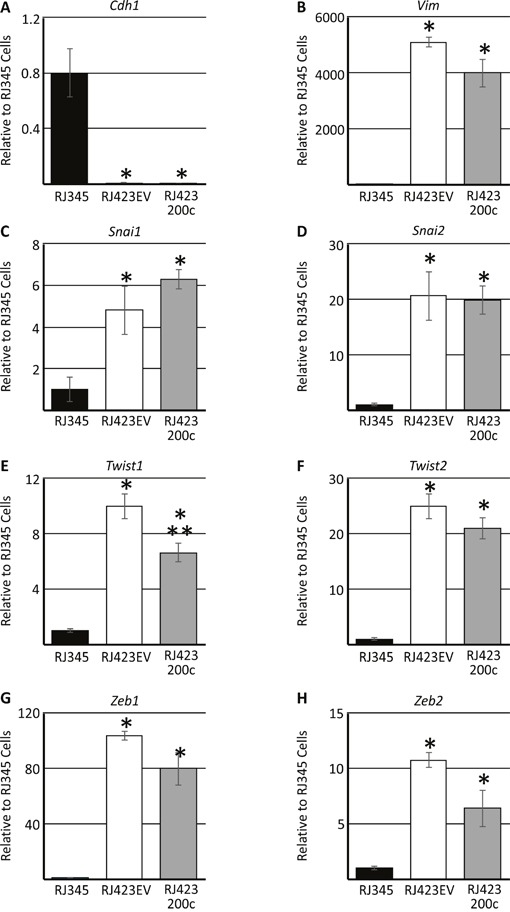
Expression (mean ± SEM) of E-cadherin/*Cdh1* **(A)**, vimentin/*Vim* **(B)**, Snail/*Snai1* **(C)**, Slug/*Snai2* **(D)**, *Twist1* **(E)**, *Twist2* **(F)**, *Zeb1* **(G)** and *Zeb2* **(H)** as determined by qRT-PCR. Gene expression in RJ423EV and RJ423200c cells are expressed relative to RJ345 cells. *Hprt* was used as the housekeeping gene and * indicates significant difference (p<0.05) in RJ4232EV and RJ423200c cells compared to RJ345 cells while ** indicates significant differences (p<0.05) between RJ423EV and RJ423200c cells. Each cell line represents n=3.

Re-expression of miR-200c in RJ423200c cells only had a modest effect on mesenchymal gene expression (Figure 5A-5H) and only *Twist1* was significantly down-regulated in RJ423200c cells compared to RJ423EV cells (Figure 5E). Zeb2 expression was approximately 40% lower in the RJ423200c cells compared to RJ423EV cells but this change did not reach significance (p=0.08). The modest change in mesenchymal gene expression was not overly surprising considering RJ423200c cells grown as monolayers retained a cell morphology more similar to RJ423 cells than RJ345 cells (Figure 1G, H).

### Re-expression of miR-200c impairs cell proliferation and anchorage independent growth but not invasion *in vitro*

**Figure 6:**
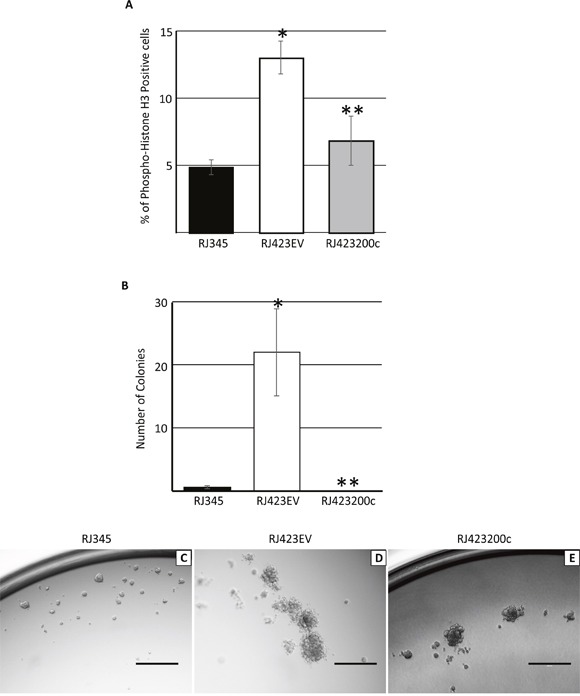
Quantification (mean ± SEM) of immunohistochemistry for phospho-histone H3 **(A)** or colony formation in soft agar **(B)** in RJ345, RJ423EV and RJ423200c cells. * indicates significant difference (p<0.05) in RJ4232EV and RJ423200c cells compared to RJ345 cells while ** indicates significant differences (p<0.05) between RJ423EV and RJ423200c cells. Each cell line represents n=3. Images in C-E are representative colonies formed by RJ345 **(C)**, RJ423EV **(D)** and RJ423200c **(E)** cells when grown as three dimensional cultures in matrigel. Scale bar, 400μm.

Proliferation was determined using phospho-histone H3 immunofluorescence in RJ345, RJ423EV and RJ423200c cells. As shown in Figure 6A, RJ423EV cells had a significantly higher percentage of cells staining positive for phospho-histone H3 than RJ345 cells. Re-expression of miR-200c in RJ423 cells significantly reduced cell proliferation compared to RJ423EV cells. These findings suggest that expression of miR-200c negatively impacts cell proliferation rates.

Apoptosis was determined using immunofluorescence for cleaved caspase 3 and there were no significant differences identified and RJ345, RJ423EV and RJ423200c cells and all cell lines had less than 0.25% of the cells stain positive for cleaved caspase 3 (data not shown).

To determine the ability of RJ345, RJ423EV and RJ423200c cells to grow under anchorage independent growth conditions these cells were grown suspended in agar. Under these conditions, RJ423EV cells produced significantly more colonies than RJ345 cells while re-expression of miR-200c in RJ423200c completely abolished the ability of RJ423 cells to produce colonies in a soft agar assay (Figure 6B).

RJ345, RJ423EV and RJ423200c cells were also grown suspended in matrigel. In matrigel, RJ345 cells formed compact, dense spheres with defined borders (Figure 6C) while RJ423EV formed were more loosely organized spheres with irregular borders (Figure 6D). RJ423200c spheres appeared to be densely packed but possessed irregular borders and thus shared characteristics with both RJ345 cells and RJ423EV cells (Figure 6E).

**Figure 7:**
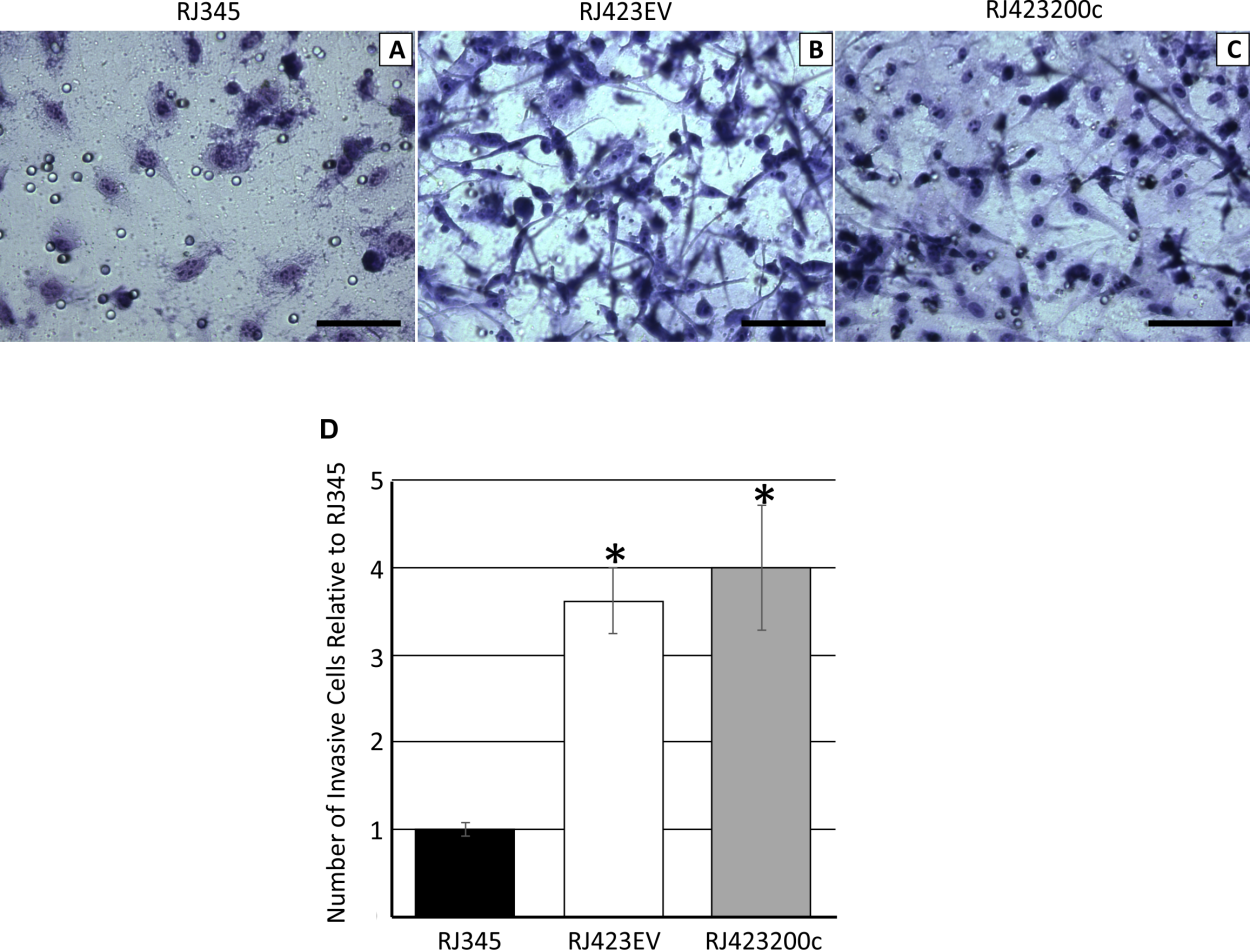
Representative images of RJ345 **(A)**, RJ423EV **(B)** and RJ423200c **(C)** cells that migrated through the invasion chamber following fixation and staining. Scale bars, 100μm. Panel **(D)** shows the quantification of the number of cells that migrated through the invasion chamber for RJ423EV and RJ42300c cells relative to RJ345 cells. The graph represents the mean and SEM of at least 3 individual trials. * indicates significant difference (p<0.05) in RJ4232EV and RJ423200c cells compared to RJ345 cells.

Cell migration was determined using an invasion chamber assay. As shown in Figure 7 significantly more RJ423EV cells migrated through the matrigel-coated invasion chambers than RJ345 cells demonstrating that the mesenchymal RJ423EV cells had superior invasive potential compared to the luminal RJ345 cells. Re-expression of miR-200c in RJ423 cells did not significantly reduce cell invasion as RJ423EV and RJ423200c cells had similar invasive properties.

### RNA sequencing

To further understand the genetic alterations induced by re-expression of miR-200c in RJ423 cells, RNA sequencing was performed on RJ423EV and RJ423200c cells using 3 independent replicates for each cell line. When the list of genes was filtered for log fold change >1 and DESeq adjusted p-value of < 0.01, 305 genes met these criteria (137 upregulated and 168 downregulated genes). The top 20 genes based on p-value are listed in Table 4.

**Table 4:** Genes differentially expressed in RJ423200c cells compared to RJ423EV cells

Gene ID	Gene Symbol	Log Fold Change^1^	Deseq Adjusted pval
ENSMUSG00000073599	*Ecscr*	−5.0	2.2×10^−55^
ENSMUSG00000070867	*Trabd2b*	4.5	5.2×10^−46^
ENSMUSG00000041078	*Grid1*	−3.2	1.0×10^−44^
ENSMUSG00000092060	*Bend4*	−7.7	2.5×10^−44^
ENSMUSG00000029810	*Tmem176b*	−8.8	3.2×10^−39^
ENSMUSG00000023367	*Tmem176a*	−9.8	1.5×10^−32^
ENSMUSG00000024598	*Fbn2*	4.3	5.1×10^−28^
ENSMUSG00000046159	*Chrm3*	−2.7	1.7×10^−26^
ENSMUSG00000025876	*Unc5a*	−2.2	4.8×10^−25^
ENSMUSG00000049404	*Rarres1*	−2.6	2.2×10^−24^
ENSMUSG00000037762	*Slc16a9*	−5.8	1.5×10^−21^
ENSMUSG00000024743	*Syt7*	−4.7	2.8×10^−21^
ENSMUSG00000060044	*Tmem26*	−2.3	8.9×10^−20^
ENSMUSG00000027797	*Dclk1*	−2.1	3.1×10^−19^
ENSMUSG00000060600	*Eno3*	−1.7	4.1×10^−19^
ENSMUSG00000036862	*Dchs1*	2.2	1.2×10^−18^
ENSMUSG00000054612	*Mgmt*	−7.1	1.6×10^−18^
ENSMUSG00000032334	*Loxl1*	1.6	3.4×10^−18^
ENSMUSG00000036585	*Fgf1*	3.9	5.6×10^−17^
ENSMUSG00000074151	*Nlrc5*	−1.8	2.5×10^−16^

^1^ negative log fold change indicate genes downregulated in RJ423200c cells relative to RJ423EV cells.

Advaita's iPathwayGuide software (Advaita Corporation, Plymouth, MI) was used to identify cellular pathways and functions associated with the differentially expressed genes. The two significant biological pathways and top 5 molecular functions identified by iPathwayGuide are presented in Table 5. This software was also used to identify genes downregulated in RJ423200c cells compared to RJ423EV cells that had miR-200c consensus sequences as predicted by iPathwayGuide software. This analysis identified 5 potential miR-200c targets and these targets are listed in Table 6. Quantitative RT-PCR confirmed that these 5 genes were significantly downregulated in RJ423200c cells compared to RJ423EV cells (Table 6).

**Table 5:** Pathways and functions identified by ipathwayguide

Biological Pathway	Pval
Transcriptional misregulation in cancer	0.043
Neuroactive ligand-receptor interaction	0.043
**Molecular Functions**	
Protein-arginine deiminase activity	0.02
Receptor binding	0.02
Receptor activity	0.02
Molecular transducer activity	0.02
Signaling receptor activity	0.02

**Table 6:** genes downregulated in RJ423200c cells with potential miR-200c target sequences

Gene ID	Gene Symbol	Log Fold Change (RNA-Seq)^1^	Deseq Adjusted pval	Log Fold Change (RT-PCR)^1^
ENSMUSG00000031355	*Arhgap6*	−1.3	2.8×10^−7^	−1.1
ENSMUSG00000045103	*Dmd*	−5.8	2.6×10^−4^	−6.6
ENSMUSG00000029648	*Flt1*	−1.2	1.7×10^−9^	−1.2
ENSMUSG00000059336	*Slc14a1*	−3.6	1.8×10^−4^	−3.9
ENSMUSG00000024924	*Vldlr*	−1.5	1.4×10^−3^	−1.3

^1^ negative log fold change indicate genes downregulated in RJ423200c cells relative to RJ423EV cells.

### Re-expression of miR-200c impairs mammary tumor growth *in vivo*

To determine whether re-expression of miR-200c in RJ423 cells affected mammary tumor growth *in vivo*, RJ423EV and RJ423200c cells were injected into the 4^th^ mammary glands of wild type, FVB mice. Two different cell numbers were injected for both RJ423EV and RJ423200c cells; 5×10^5^ and 5×10^4^ cells per mammary gland. Tumor development was similar following injection of either 5×10^5^ or 5×10^4^ cells and thus, the data from the different cell numbers was pooled. Injection of RJ345 cells was not performed as our previous studies demonstrated that RJ345 cells are only weakly tumorigenic (only 50% of the mammary gland injected with 2.5×10^6^ RJ345 cells produced tumors and injection of 5×10^5^ RJ345 cells failed to produce mammary tumors [40]). In contrast, 100% of the injections with either 5×10^5^ or 5×10^4^ cells for either RJ423EV or RJ423200c cells produced mammary tumors.

**Figure 8:**
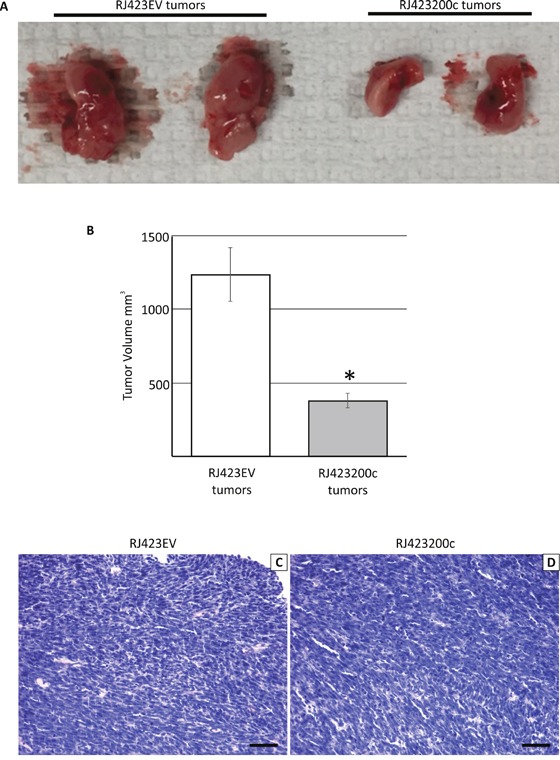
Representative macroscopic images of the mammary tumors that developed following the injection of RJ423EV or RJ423200c cells **(A)**. Tumor volume (mean ± SEM) was determined and was plotted as a bar graph **(B)**. * indicates significant difference (p<0.05). Panels C and D show representative hematoxylin and eosin stained sections of tumors induced following injection of RJ423EV **(C)** or RJ423200c **(D)** cells. Scale bars, 100μm.

The images in Figure 8A show representative tumors following injection of RJ423EV and RJ423200c cells. All tumors were collected when the RJ423EV tumors reached approximately 10% of the mouse's body weight which is the maximum tumor size allowed by the Canadian Council for Animal Care. As shown in Figure 8B, tumor volume was significantly lower in the mammary tumors induced by injection of RJ423200c cells compared to those produced by injection of RJ423EV cells (Figure 8B).

Tumors produced by the injection of either RJ423EV or RJ423200c cells had similar histologic features (Figure 8C, 8D). The tumors were composed primarily of densely packed tumor cells with little stroma and frequently displayed regions of necrosis.

**Figure 9:**
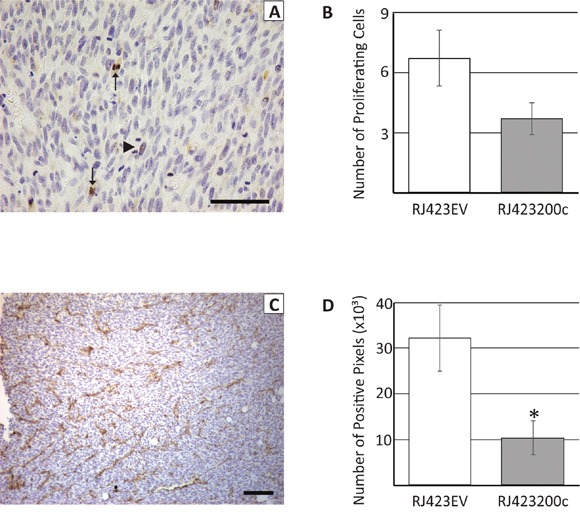
Representative section of a tumor stained with anti-Ki67 **(A)** or anti-CD31 **(C)**. Arrows in panel (A) indicate positive staining for Ki67 while the arrowhead indicates a mitotic cell that did not stain with the anti-Ki67 antibody. Ki67 and CD31 staining were quantified and this data is shown in panels **(B)** and **(D)**, respectively. Each bar represents the mean ± SEM of 3 independent trials, *p<0.05. Scale bars, 100μm.

In an attempt to understand the mechanism of reduced tumor growth induced by miR-200c, proliferation, apoptosis and blood vessel density were assessed. To identify proliferating tumor cells, Ki67 immunohistochemistry was performed. Analysis of the Ki67 stained sections revealed that proliferating tumor cells were not evenly distributed throughout the tumor and cells undergoing anaphase were not stained by Ki67 (Figure 9A; Ki67 expression decreases during anaphase and telophase [48, 49]). Thus, in an attempt to accurately capture tumor cell proliferation rate, 5 sections of each tumor were randomly selected and the number of Ki-67 positive and anaphase cells were counted in a blinded manner (Figure 9A). The number of proliferating cells were then averaged across the 5 fields and we found that there was a non-significant (p=0.089), 1.8-fold decrease in tumor cell proliferation in RJ423200c tumors compared to RJ423EV tumors (Figure 9B).

Cleaved caspase-3 immunohistochemistry was performed to evaluate tumor apoptosis. However, a number of the tumors contained large regions of apparent necrosis and these regions stained strongly for cleaved caspase 3 (data not shown). These regions of staining made it impossible to accurately compare apoptosis between RJ423EV and RJ423200c tumors.

During tumor collection it was observed that mammary tumors induced by RJ423200c cells appeared to have fewer visible blood vessels compared to mammary tumors induced by RJ423EV cells. Therefore, blood vessel density was evaluated using CD31 immunohistochemistry. CD31 staining was variable in both RJ423200c and RJ423EV tumors with higher levels typically observed near the tumor periphery compared to the interior regions of the tumor. In an attempt to accurately determine whether differences in CD31 staining existed between RJ423EV and RJ423200c tumors, four images were captured for each tumor; two areas of highest CD31 staining and two areas of lowest CD31 staining. The amount of CD31 staining was quantified using ImageScope positive pixel count v9.1 software (Aperio Technologies Inc, Vista, CA) and the average staining for the 4 images from each tumor was determined. As shown in Figure 9C, 9D the amount of CD31 staining was significantly higher in the tumors produced by RJ423EV cells compared to tumors produced by RJ423200c cells.

## DISCUSSION

Our previous work demonstrated that mammary tumors induced by overexpression of IGF-IR in MTB-IGFIR transgenic mice, also know as primary mammary tumors or PMTs, had features of luminal tumors such as an epithelial morphology and E-cadherin expression, however, these tumors clustered most closely with human basal-like breast tumors [39]. PMTs do not express the estrogen receptor (ER), as determined by immunohistochemistry, and this likely reduces the similarity of their gene expression profile to human luminal tumors which express ER and have high levels of ER regulated genes. In contrast, recurrent spindle tumors or RSTs develop in a subset of mice following downregulation of the IGF-IR. RSTs have gene expression patterns similar to human claudin-low breast cancers [39]. Claudin-low breast cancer is a relatively rare breast cancer subtype where the tumor cells possess mesenchymal characteristics as well as very low levels of claudin-3 (*Cldn3*), *Cldn4* and *Cdln7*; properties that were observed in the RSTs [31, 32]. At least 2 new classification systems have been described for breast cancer and neither of these systems utilize the term claudin-low. In the classification system proposed by Lehmann et al [34], RSTs would be classified as mesenchymal stem-like tumors and in the system proposed by Jézéquel et al [35] RSTs would be classified as basal-enriched with high immune response and low M2 macrophage subtype. To be consistent with our previous publications the term claudin-low has been used in this study to describe tumors and cell lines derived from MTB-IGFIR mice that possess a mesenchymal morphology and express high levels of mesenchymal genes and low levels of E-cadherin and claudins-3, -4, -7.

A miRNA array was utilized to investigate miRNAs differentially expressed in PMTs compared to RSTs. A number of miRNAs were identified including the miR-200f where all 5 members of this miRNA family were expressed at significantly lower levels in RSTs compared to PMTs. Reduced miR-200f expression in mesenchymal mammary tumors is consistent with studies published by Castilla et al [50] who demonstrated that luminal breast cancer had the highest level of miR-200f while mesenchymal breast cancers such as metaplastic breast cancer expressed only very low levels of miR-200f and Herschowitz et al [51] who demonstrated that murine mammary tumors with features of claudin-low breast cancer presented with the lowest expression of miR-200f when compared to murine mammary tumors with features of other breast cancer subtypes. Moreover, a number of studies have demonstrated that one of the main functions of the miR-200f is to maintain cells in an epithelial state, at least in part, through the regulation of mesenchymal genes such as *Snai1*, *Snai2*, *Twist1*, *Twist2*, *Zeb1* and *Zeb2* (reviewed in [23, 24, 52–61]). Consistent with the low levels of the miR-200f in RSTs, these tumors express high levels of mesenchymal genes.

As it is difficult to manipulate miRNAs in transgenic mice, cell lines were derived from PMTs and RSTs and it was shown that these cell lines maintain gene and miRNA expression patterns similar to the tumors from which they were derived. RJ345 cells were derived from a PMT and these cells maintain an epithelial morphology in culture, express *Cdh1* and only low levels of mesenchymal genes. RJ348 and RJ423 cells were derived from two different RSTs and both of these lines possess a mesenchymal morphology in culture, only express very low levels of *Cdh1* but express higher levels of mesenchymal genes than the RJ345 cells. In addition, RJ345 cells express significantly higher levels of all members of the miR-200f compared to RJ348 and RJ423 cells.

In order to evaluate the function of the miR-200f in mesenchymal mammary tumor cells, miR-200c was re-expressed in RJ423 cells. This study focused on miR-200c as it was easier to manipulate a single miRNA rather than one or both miR-200f clusters and regulating an individual miRNA would simplify the analysis as each miRNA can potentially regulate hundreds or even thousands of mRNAs. Our study focused on the RJ423 cells as these cells are easier to stably transfect than the RJ348 cells.

Using a plasmid containing mmu-miR-200c driven by a CMV promoter, mature miR-200c levels could be restored nearly to the level observed in RJ345 cells and >300-fold higher than RJ423 cells transfected with an empty vector (RJ423EV cells). The expression of the other miR-200f members was not affected by transfection with the CMV-miR200c plasmid. Re-expression of miR-200c in RJ423 cells significantly reduced cell proliferation *in vitro*, however, the proliferation rate was not restored to the levels observed in RJ345 cells. Our findings are consistent with the study by Song et al [62] who demonstrated that miR-200c can suppress mammary tumor cell proliferation.

A number of studies have shown that miR-200f members negatively regulate cell migration and/or invasion [63–74] and we found that RJ423EV cells, which have very low miR-200f expression and high *Zeb1*/*Zeb2* expression, were more invasive that RJ345 cells which have low *Zeb1*/*Zeb2* expression and high miR-200f expression. However, re-expression of miR-200c in RJ423 cells did not significantly alter cell invasion. The lack of change in invasive properties following miR-200c re-expression in our study could be due to the modest changes in several of the mesenchymal genes such as *Zeb1* and *Zeb2* that have also been implicated in regulating cell migration and invasion [75–79]. Neither *Zeb1* nor *Zeb2* were significantly reduced by the re-expression of miR-200c in RJ423 cells. It should however be noted that one recent manuscript reported that re-expressed the miR-141/200c cluster in the human claudin-low breast cancer cell line, MDA-MB-231, found that miR-141/200c re-expression was associated with increased migration [80]. Therefore, it remains unclear whether miR-200f members universally inhibit tumor cell migration and/or invasion in all cell lines or whether the levels of miR-200f expression, the cell lines used, or the conditions employed can influence whether miR-200f can inhibit or stimulate migration and invasion.

Cell morphology displayed by RJ423EV and RJ423200c cells grown as monolayers was similar suggesting the re-expressing only miR-200c was insufficient to convert the mesenchymal RJ423 cells to epithelial cells. Re-expression of miR-200c did however induce a partial reversion of sphere morphology when the cells were grown as three dimensional cultures in matrigel. RJ423EV cells produced very loosely packed spheres similar to those observed for MDA-MB-231, a human claudin-low cell line while RJ345 cells produced tightly packed spheres similar to human luminal breast cancer cell lines [81]. RJ423200c spheres appeared to be densely packed but did not have the same clearly demarcated borders observed in RJ345 spheres.

One property that was completely restored was the inability to grow in soft agar. RJ423EV cells were capable of forming colonies under anchorage independent conditions while RJ345 cell very rarely formed colonies and RJ423200c failed to form colonies under these conditions. Although anchorage independent growth is only one characteristic of stem/progenitor cells, this finding is consistent with studies demonstrating that miR-200f members, including miR-200c can negatively influence cancer stem cell function [17, 18, 25, 65, 82, 83]. One specific example is a study by Shimono et al [18] that compared miRNA profiles between breast cancer stem cells and the remaining non-tumorigenic, breast cancer cells and 3 miRNA clusters, miR-200c/141, miR-200b/200a/429 and miR-183/96/182, were consistently downregulated in breast cancer stem cells [18]. These 3 miRNA clusters were also found to be downregulated in normal mouse mammary stem/progenitor cells [18]. In addition, re-expression of miR-200c into MMTV-Wnt-1 induced murine mammary tumor cells almost completely suppressed colony formation and re-expression of miR-200c in human breast cancer stem cells almost completely inhibited *in vivo* tumor growth in NOD/SCID mice [18, 82]. Therefore, our study and others suggest that miR-200c can inhibit progenitor/stem cell function in mammary tumor cells.

Other potential contributors of reduced *in vivo* tumor growth were the observed 1.8-fold decrease in tumor cell proliferation (although this difference did not reach statistical significance; p=0.089) and a significant decrease in CD31 staining. CD31 is a transmembrane glycoprotein found in endothelial cells, that is used to detect tumor vasculature [84–86]. Interestingly, one of the genes regulated by miR-200c and found to be significantly downregulated in RJ42300c cells was *Flt1* [87]. FLT1 also known as VEGFR1 (vascular endothelial growth factor receptor 1) is a tyrosine kinase receptor that binds VEGF-A, VEGF-B and placental growth factor (PLGF) and regulates angiogenesis in normal tissues as well as tumors (reviewed in [88, 89]). However, FLT1 traditionally regulates angiogenesis through it's function in endothelial cells, not tumor cells, so it remains unclear how tumor cell *Flt1* expression could influence tumor vascularity. *Flt1* expression has been found in tumor cells including breast cancer cells [90, 91] where FLT1 functions as a tumor growth regulator and pro-survival factor. Other members of the *Vegf* family were evaluated in our RNA sequencing data and only *Vegfc* was significantly downregulated in RJ423200c cells compared to RJ423EV cells. Although iPathwayGuide did not identify *Vegfc* as a miR-200c target, *Vegfc* does contain a predicted miR-200c binding site (microRNA.org) and thus can potentially be regulated by miR-200c expression. Therefore, decreased *Flt1* expression in the RJ423200c cells may suppress proliferation and reduced *Vegfc* expression in RJ423200c cells could restrict angiogenesis, both of which could contribute to the observed reduction in tumor growth. Inhibition of angiogenesis by miR-200c is consistent with a study by Pecot et al [92] that showed miR-200 family members could regulate tumor angiogenesis in basal-like breast cancer.

Other miR-200c targets identified by RNA sequencing included *Arhgap6*, *Dmd*, *Slc14a1*, and *Vldlr*. *Dmd* and *Vldlr* have previously been identified as regulators of breast cancer where their expression has been associated with paclitaxel or bevacizumab response [93, 94] and migration/metastasis [95]. *Arhgap6* and *Slc14a1* have not been implicated in breast cancer but have been implicated in a number of other cancers including cervical, endometrial, prostate and bladder cancer [96–100]. Interestingly, bevacizumab, an antibody that inhibits VEGFA function and thus angiogenesis, altered the expression of *Vldlr* in basal-like breast cancer [93] and *Arhgap6* in endometrial cancer [97]. Therefore, *Vldlr* and *Arhgap6* may also impact tumor angiogenesis in our model.

In summary, re-expression of miR-200c in murine claudin-low mammary tumor cells reduces proliferation and colony formation *in vitro* and tumor growth *in vivo*. Thus, our work further demonstrates the importance of restoring miR-200c expression as a mechanism to inhibit the growth/tumorigenic potential of claudin-low mammary tumor cells.

## MATERIALS AND METHODS

### miRNA Array

One hundred micrograms of total RNA from each sample was sent to the University Health Network Microarray Centre, Toronto, ON, Canada and RNA quality check, labeling, hybridization and scanning were performed by the University Health Network Microarray Centre. RNA quality was checked using an Agilent Bioanalyzer (Agilent Technologies, Santa Clara, CA) and all samples had a RIN > 7. RNA was then labelled and hybridized using Agilent miRNA labeling and hybridization kits following the manufacturer's protocols and miRNA expression was determined using an Agilent 8×15K v2:627 mouse miRNA array (Agilent Technologies, Santa Clara, CA). Slides were scanned on a G2565C scanner (Agilent Technologies, Santa Clara, CA) and values were generated using feature extraction software version 10.5 and the extraction protocol miRNA_105_Dec08. Fold changes in miRNAs was determined using Genespring 12 (Agilent Technologies, Santa Clara, CA).

### RNA extraction, taqman qRT-PCR for microRNA expression and qRT-PCR for gene expression

RNA was extracted using a mirVana miRNA Isolation kit (Thermo Fisher Scientific, Burlington, ON). For the analysis of miRNA expression, the TaqMan microRNA reverse transcription kit (Thermo Fisher Scientific, Burlington, ON) was use to reverse transcript 100 ng of total RNA into cDNA following the manufacturer's protocol. Briefly, 100 ng of RNA (in 5 μl) was mixed with 0.15μl of dNTP mix (100mM total), 1 μl multiscribe RT enzyme (50U/μl), 1.5 μl of 10xRT buffer, 0.19 μl of RNase inhibitor (20U/μl), 4.16 μl of nuclease-free water and 3 μl of 5x miRNA primer. Reverse transcription was performed on a thermal cycler with samples incubated at 16°C for 30min, 42°C for 30min, 85°C for 5min and then held at 4°C until used for qPCR. Primers for reverse transcribing (5x primers) and for amplifying cDNA (20x primers) for miR-141 (ID 000463), miR-200a (ID 000502), miR-200b (ID 001800), miR-200c (ID 002300), miR-429, SnoRNA202 (ID 001232) and SnoRNA234 (ID 001234) were obtained from Thermo Fisher Scientific (Burlington, ON). qPCR was performed a CFX96 real-time PCR machine (Bio-Rad Laboratories, Mississauga, ON) using 2μl of each cDNA reaction, 10ul of TaqMan universal master mix II no UNG (Thermo Fisher Scientific, Burlington, ON) 1μl of 20x primer and 7μl of water and the following incubation conditions; 50°C for 2min, 95°C for 10min and then 40 cycles of 95°C for 15s and 60°C for 1min. miRNA levels were calculated using Bio-Rad CFX Manager 3.1 (Bio-Rad Laboratories, Mississauga, ON) and were normalized to the levels of SnoRNA202 and SnoRNA234.

For the analysis of gene expression, 1 ug of total RNA was reverse transcribed using 4ul of qScript cDNA supermix (Quanta Biosciences, Beverly, MA) per 20μl reaction and the following incubation conditions on a thermal cycler; 25°C for 5min, 42°C for 30min, 85°C for 5min and then held at 4°C until used for qPCR. For qPCR, 1μl of cDNA was mixed with 10μl of SsoFast EvaGreen supermix (Bio-Rad Laboratories, Mississauga, ON) and 9μl of water and gene expression was quantified using a CFX96 real-time PCR machine (Bio-Rad Laboratories, Mississauga, ON) and the following program; 95°C for 2min and then 40 cycles of 95°C for 5s and 60°C for 30s. All gene primers were obtained from Bio-Rad Laboratories (Mississauga, ON); *Arhgap6* (aMmuCID0009097), *Cdh1* (qMmuCID0005843), *Cldn3* (qMmuCED0001019), *Cldn4* (qMmuCED0003218), *Cldn7* (qMmuCED0005006), *Dmd* (qMmuCID0018306), *Flt1* (qMmuCID0016762), *Hprt* (qMmuCED0045738), *Slc14a1* (qMmuCID0017253), *Twist1* (qMmuCED0004065), *Twist2* (qMmuCID0009652) *Vim* (qMmuCID0005527), *Vldlr* (aMmuCID0011365), *Zeb1* (qMmuCID0009095), and *Zeb2* (qMmuCID0014662). Gene expression was quantified using Bio-Rad CFX Manager 3.1 (Bio-Rad Laboratories, Mississauga, ON) and were normalized to the levels of *Hprt*.

### miR-200c promoter methylation analysis

Two approaches were utilized to determine methylation of the miR-200c promoter. In the first approach, DNA isolated from RJ345, RJ423 and RJ348 cells using a Quick-DNA Universal Kit (Cedarlane, Burlington, ON) or DNA isolated from primary mammary tumors (PMTs) or recurrent spindle tumors (RSTs), described in [41], using DNeasy Blood and Tissue Kit (Qiagen, Germantown, MD) was sent to Zymo Research Corporation for targeted bisulfite sequencing of the miR-200c/141 promoter region. DNA samples sent to Zymo Research were bisulfite converted and relevant regions were PCR amplified using 6 sets of primers covering 33 CpG site in the first 1000bp upstream of the miR-200c/141 cluster. The PCR amplified DNA was then sequenced. A CpG methylation ratio was calculated by determining the number reads of methylated CpG sites relative to the total CpG read counts. Only CpG sites with at least 10 reads were evaluated and this meant that 30 CpG sites were evaluated.

In the second approach, RJ423 cells were treated with 3μM of 5-aza-2′-deoxycytidine daily for 72 hours or the equivalent amount of DMSO daily for 72 hours. At the end of the treatment period, RNA was extracted and Taqman qRT-PCR for miR-200c or miR-200b (first member of each cluster) was performed as described above using SnoRNA202 and SnoRNA234 as normalizers.

### Expression plasmids and generation of stable cell lines

The microRNA expression plasmid containing murine miR-200c was purchased from Origene (cat # SC400903; Origene Technologies, Rockville, MD). In this plasmid, mmu-miR-200c expression is driven by a CMV promoter. RJ423200c cells were created by transfecting RJ423 cells with 1μg of the pCMV-miR-200c plasmid using 5μl/ml of Lipofectamine 2000 (Thermo Fisher Scientific, Burlington, ON). The same conditions were used to transfect RJ423 cells with the empty vector control (cat# PCMVMIR; Origene Technologies, Rockville, MD). Cells were cultured in the presence of 1 mg/ml Geneticin (Thermo Fisher Scientific, Burlington, ON; this concentration was sufficient to kill untransfected, parental cells).

### Immunofluorescence

Cell proliferation was determined using immunofluorescence for phospho-histone H3 while apoptosis was determined using immunofluorescence for cleaved caspase 3. 1 × 10^5^ of 345, RJ423EV or RJ423200c cells were each plated in fully supplemented media on sterile glass coverslips in a 6-well plate. The next day, cells were washed in PBS and fixed in 10% neutral buffered formalin for 1 hour at room temperature. The cells were then washed again with PBS and permeabilized with 0.2% Triton X-100 in PBS solution for 5 minutes. Cells were then blocked using 5% BSA, 0.1% Triton X-100 in PBS for 10 minutes. Coverslips were incubated with either a 1:2,000 dilution of anti-phospho histone H3 (Abcam Inc, Toronto, ON cat#ab14955) or a 1:500 dilution of anti-cleaved caspase 3 (Cell Signaling Technology, MA cat#ab9661) overnight at 4°C. The coverslips were then incubated with a 1:500 dilution of goat anti-mouse Alexa Fluor 488 secondary antibody (Thermo Fisher Scientific, Burlington, ON cat#A-11001) at room temperature for 1 hour. Cell nuclei were counterstained with DAPI and mounted using Prolong Gold (Thermo Fisher Scientific, Burlington, ON). Images were taken using MetaMorph Imaging Software (Molecular Devices, Sunnyvale, CA) on an Olympus BX961 fluorescent signal microscope (Olympus, Center Valley, PA). The percentage of cells positive for phospho-histone H3 or cleaved caspase 3 was determined by manual counting using the ImageJ software (National Institutes of Health, Bethesda, MD). Experiments were performed in triplicate (n=3).

### Immunohistochemistry

To detect blood vessels, immunohistochemistry was performed as previously described [101] using a 1:100 dilution of anti-CD31 (cat #77699; Cell Signaling, Danvers, MA) in 5% goat serum in tris-buffered saline containing 0.1% Tween 20. Images were captured on a Nikon E600 microscope (Nikon Instruments Inc, Melville, NY). For each tumor, two regions of the highest levels of CD31 staining and two regions of the lowest levels of CD31 staining were captured and the amount of staining was quantified using the positive pixel count v9 algorithm in ImageScope software (Aperio Technologies, Vista, CA) and the average staining per tumor was determined.

To detect the number of proliferating tumor cells, immunohistochemistry was performed as previously described [101] using a 1:200 dilution of Ki67 (cat#12202; Cell Signaling, Danvers, MA) in 5% goat serum in tris-buffered saline containing 0.1% Tween 20. Each tumor section was analyzed in a blinded manner and 5 fields were randomly selected. In each field, the number of Ki67 positive cells and the number of mitotic figures were counted and the number counted in the 5 fields were averaged to obtain the number of proliferating cells/field/tumor.

### Invasion chamber assay

Invasion chamber inserts from BioCoat Matrigel Invasion Chamber kit (Corning, NY) were used. The inserts were thawed and rehydrated in a 24 well plate using serum free media both beneath and inside the insert for 2 hours. The inserts were placed on top of 750μl of serum containing media in a new well of the 24 well plate. 5 × 10^4^ of RJ345, RJ423EV, or RJ423200c cells were each seeded in serum free media on top of the inserts. After 24 hours, cells that had migrated to the opposite sided of the chamber were fixed in 100% methanol for 2 minutes at room temperature and then stained with toluidine blue for 2 minutes at room temperature. Cells were imaged using an inverted Olympus IX71 microscope with Q Imaging software (Q Imaging). The number of cells able to migrate and invade through the insert were counted manually using the ImageJ software (National Institutes of Health). Experiments were performed in triplicate (n=3).

### Soft agar assay

A 4mL layer of 1:1, 1.5% agar and 2X media was placed in a 60ml dish and allowed to solidify for 30 minutes to prevent the attachment of cells to the bottom of the dish. After the agar had solidified 2 × 10^4^ of RJ345, RJ423EV, or RJ423200c cells were seeded in a 1:1 mixture of 2X media and 0.75% agar solution on top of the base agar layer in the 60ml dish. At least 3 plates were seeded for each cell line and the experiment was repeated on three different days. Media was added twice weekly for 30 days. After 30 days, individual colonies were manually counted.

### 3D Culture in matrigel

Cell lines were seeded in 150ul of growth media supplemented with 5% growth-factor reduced Matrigel® (Thermo Fisher Scientific, Burlington, ON) at a density of 500 cells per well in 96-well low attachment plates (Thermo Fisher Scientific, Burlington, ON). Cells were cultured up to 7 days without further media changes. Images were acquired using the EVOS Cell Imaging System (Thermo Fisher Scientific, Burlington, ON).

### RNA sequencing

RNA sequencing and analysis was performed on RNA extracted from RJ423EV and RJ423200c cells at the Genome Quebec Innovation Centre at McGill University using the Illumina Hiseq 2500 v4 PE125. Reads were trimmed using Trimmomatic software [102] from the 3′ end to have a phred score of at least 30. Sequencing adapters were removed from the reads and only reads of at least 32 base pairs were used. Filtered reads were aligned to the Mus_musculus assembly GRCm38 reference genome using STAR [103]. RNA-seq reads were aligned to genes and their abundance estimated using Cufflinks [104]. Differential gene expression analysis was performed using DESeq [105] and edgeR [106] R Bioconductor packages. RNA Sequencing data was further analyzed with iPathwayGuide software (Advaita Corporation, Plymouth, MI) using genes with at least 2-fold difference in expression and a minimum Deseq adjusted p-value of 0.01. For pathway analysis in iPathwayGuide, FDR correction was used.

### Intra-mammary tumor cell injection

Tumor cells were injected into the mammary glands of wild type, syngeneic, FVB mice as described in [107] using 5×10^4^ or 5×10^5^ cells. All mice were monitored 2 times per week by palpating the mammary glands. Once a palpable mammary tumor was identified tumor size was measured using digital calipers. Once the RJ423EV tumors reached ~10% of the mouse's body weight, the mice bearing RJ423EV or RJ423200c tumors were euthanized and the mammary tumors collected. Each mammary tumor was divided for fixation in formalin, cryopreservation in OCT and flash frozen in liquid nitrogen. Animals were housed and cared for following guidelines established by the Central Animal Facility at the University of Guelph and the guidelines established by the Canadian Council of Animal Care. This study was approved by the Animal Care Committee at the University of Guelph.

### Statistics

For analysis comparing the means of two different groups, a Student's t-test was performed. For analyses comparing the means of three or more groups, an ANOVA followed by a Tukey's test was performed. In both cases, means were considered statistically different when p < 0.05.

## Abbreviations

Arhgap6: Rho GTPase activating protein 6
CD31: platelet and endothelial cell adhesion molecule 1
Cdh1: E-cadherin
Cldn: claudin
CMV: cytomegalovirus
Dmd: dystrophin
EMT: epithelial-to-mesenchymal transition
ER: estrogen receptor
Flt1: Fms related tyrosine kinase 1
HER2: human epidermal growth factor receptor 2
Hprt: hypoxanthine phosphoribosyltranferase 1
IGF-IR: type I insulin-like growth factor receptor
Ki67: marker of proliferation KI-67
miR: microRNA
miR-200f: miRNA-200 family
MTB-IGFIR: transgenic mice expressing IGF-IR in mammary epithelial cells
nt: nucleotide
PMT: primary mammary tumor
PR: progesterone receptor
RISC: RNA-induced silencing complex
RST: recurrent spindle tumor
S100a4: S100 calcium binding protein 4
Slc14a1: solute carrier family 14 member 1 (Kidd blood group)
Snai1: snail family transcriptional repressor 1
Snai2: snail family transcriptional repressor 2
Twist1: twist family BHLH transcription factor 1
Twist2: twist family BHLH transcription factor 2
UTR: untranslated region
Vegfc: vascular endothelial growth factor c
Vim: vimentin
Vldlr: very low density lipoprotein receptor
Zeb1: zinc finger E-box binding homeobox 1
Zeb2: zinc finger E-box binding homeobox 2
